# Cellograph: a semi-supervised approach to analyzing multi-condition single-cell RNA-sequencing data using graph neural networks

**DOI:** 10.1186/s12859-024-05641-9

**Published:** 2024-01-15

**Authors:** Jamshaid A. Shahir, Natalie Stanley, Jeremy E. Purvis

**Affiliations:** 1 Department of Genetics, University of North Carolina at Chapel Hill, Chapel Hill, NC USA; 2 Curriculum in Bioinformatics and Computational Biology, University of North Carolina at Chapel Hill, Chapel Hill, NC USA; 3 Computational Medicine Program, University of North Carolina at Chapel Hill, Chapel Hill, NC USA; 4 Department of Computer Science, University of North Carolina at Chapel Hill, Chapel Hill, NC USA; 5 Lineberger Comprehensive Cancer Center, University of North Carolina at Chapel Hill, Chapel Hill, NC USA

**Keywords:** Graph neural networks, Single-cell genomics, Semi-supervised learning

## Abstract

With the growing number of single-cell datasets collected under more complex experimental conditions, there is an opportunity to leverage single-cell variability to reveal deeper insights into how cells respond to perturbations. Many existing approaches rely on discretizing the data into clusters for differential gene expression (DGE), effectively ironing out any information unveiled by the single-cell variability across cell-types. In addition, DGE often assumes a statistical distribution that, if erroneous, can lead to false positive differentially expressed genes. Here, we present Cellograph: a semi-supervised framework that uses graph neural networks to quantify the effects of perturbations at single-cell granularity. Cellograph not only measures how prototypical cells are of each condition but also learns a latent space that is amenable to interpretable data visualization and clustering. The learned gene weight matrix from training reveals pertinent genes driving the differences between conditions. We demonstrate the utility of our approach on publicly-available datasets including cancer drug therapy, stem cell reprogramming, and organoid differentiation. Cellograph outperforms existing methods for quantifying the effects of experimental perturbations and offers a novel framework to analyze single-cell data using deep learning.

**Supplementary Information:**

The online version contains supplementary material available at 10.1186/s12859-024-05641-9.

## Background

The rapid progression of single-cell technologies [1] has enabled scientists to accumulate complex datasets to study differentiation and developmental trajectories in response to differing experimental perturbations, assess the efficacy of a drug in treating of disease, and evaluate the efficiency of different reprogramming protocols. Regardless of the preceding experimental design, many single-cell RNA sequencing (scRNA-seq) analyses follow the same pipeline [2]: pre-processing and quality control followed by clustering and differential gene expression. In the context of studying more continuous phenomena such as differentiation or cell reprogramming, trajectory analysis may also be employed [3]. However, in the case of multiple experimental conditions such as different time points sampled for sequencing in cell reprogramming, or varying concentrations of a cancer drug, these methods may fall short in faithfully summarizing the underlying biology. In particular, clustering and differential gene expression give a bulk summary of the transcriptomic variation between computationally-inferred discrete populations, but do not explicitly consider the single-cell variability within treatment groups, such as how prototypical an individual cell is of its assigned treatment group.

### Related methods

Differential abundance methods can rectify these challenges by quantifying differences between and within conditions at a finer resolution. Milo tests for differential abundance on *k*-nearest neighbor (*k*NN) graphs by aggregating cells into overlapping neighborhoods and performing a quasi-likelihood F test [4]. This returns a metric of the log-fold change of the differential abundance in each neighborhood. However, because Milo aggregates cells into neighborhoods, it does not provide single-cell resolution providing insight into the impact each perturbation has on an individual cell.

Covarying Neighbor Analysis (CNA) [5] performs association analysis agnostic of parameter tuning, making it an efficient method. Like Milo, it aggregates cells into neighborhoods, and calculates a neighborhood abundance matrix (NAM), where each entry $C_{n,m}$ is the relative abundance of cells from sample *n* in neighborhood *m*. From there, it derives principal components where positive loadings correspond to higher abundance while negative loadings correspond to lower abundance. This enables the characterization of transcriptional changes corresponding to maximal variation in neighborhood abundance across samples. Association testing is performed between transcriptional changes and attributes of interest using the first *k* NAM-PCs. It returns the Spearman correlation between the attribute and abundance of the neighborhood anchored at each cell, providing a single-cell metric. However, its performance falls short when considering more than two conditions.

MELD [6] sought out to quantify the effect of an experimental perturbation on individual cells in scRNA-seq data using graph signal-processing to infer a sample-associated density that is then normalized to give a probability of each cell belonging to a condition of interest defined as a relative likelihood. It uses all the class labels to derive these probabilities. The authors also introduced a novel clustering approach, called Vertex Frequency Clustering (VFC), which clusters data according to not just transcriptomic similarity but also how the MELD-derived relative likelihood scores, thereby identifying populations of cells similarly enriched or depleted in conditions according to the perturbation response. However, the original study restricted evaluation to datasets with two conditions to discriminate between: a control condition and a single perturbed condition, and therefore did not consider multiple treatment conditions, which are more prevalent and can provide more insight, for instance, the response of a drug at various time intervals, combining drugs, or administration of a differentiation stimulus at different time-points. Furthermore, robust calculation of the sample-associated likelihood relies on computationally-expensive parameter estimation that can take upwards of 12 h with 36 cores on a high-performance computing cluster for a dataset of 26,827 cells. In addition, VFC is memory-intensive, which limits its scalability to larger datasets.

### Graph neural networks

In recent years, the rapidly-emerging field of deep learning has seen utility in scRNA-seq analysis [7–9]. More recently, graph neural networks (GNN) have demonstrated promise in capturing the structural information of scRNA-seq data via the graphical representation the high-dimensional assay naturally lends itself towards, with cells as vertices or nodes, and edges between them representing similarity in gene expression. This connectivity enables the model to naturally leverage the relationship between similar cells in a variety of tasks, most notably clustering and imputation. GNNs pass in graphical representations of data as input to perform a myriad of classification tasks, namely node classification, edge classification, and graph classification. Unlike convolutional neural networks (CNNS) which involve multiple layers and can take a long time to train depending on the size of the data, GNNs require only a few layers to achieve high performance in a fraction of the time. Furthermore, whereas CNNs require large amounts of training data, GNNs can learn patterns in data in a semi-supervised fashion: they take the entire data structure as input, but only a paucity of nodes are labeled; a larger portion is held out for validation and testing purposes. The applications of GNNs has been demonstrated in the case of graph classification, edge classification, and node classification. For example, scGNN [9] used GNNs and a Gaussian mixture model to perform imputation and cell clustering. Another study used Graph Attention Networks (GATs) [10, 11]—a subset of GNNs based on the self-attention mechanism commonly used in natural language processing—to predict disease state in scRNA-seq data from multiple sclerosis patients, followed by another study from the same group applied to COVID-19 patients [12]. GATs have also been used as part of variational graph autoencoders to facilitate clustering [13].

Moreover, GNNs have been used in conjunction with relational networks to predict breast cancer subtypes in bulk RNA-seq data [14]. However, their potential to ascertain the responsiveness of individual cells to perturbations in order to gauge the efficacy of the experimental stimulus, particularly in complex experimental designs that span multiple conditions or time points, has not been formally assessed.

Finally, a notable study introduced a GNN framework called single-cell Graph Convolutional Network (scGCN) which uses Graph Convolution Networks (GCNs) [15]—which are analogous to CNNs in that they both use convolution operators, but operate on different types of data structures—to transfer labels across diverse datasets and subsequently integrate the datasets, outperforming popular methods like Seurat v3 and Conos on these tasks [16]. However, the framework could not perform perturbation analysis, as its task was to predict cell type annotations of query data from the given reference data, illustrating room for the expansion of the novel applications of GNNs in single cell genomics, to which our work below seeks to contribute.

In this work, we introduce Cellograph: a novel computational framework using GCNs to perform node classification on scRNA-seq data collected from multiple conditions, treating the individual cells as nodes. Cellograph uses a two-layer GCN to learn a latent representation of the single-cell data according to how representative each cell is of its ground truth sample label. This latent space can be easily clustered to derive groups of cells associated with similar treatment response and transcriptomics, as well as projected into two dimensions for visualization purposes. Cellograph outperforms existing approaches in quantifying the effects of perturbations and offers a novel GNN framework to cluster and visualize single-cell data. In addition, Cellograph is more scalable, performing at least an order of magnitude faster than MELD. In the following sections, we discuss the workflow of Cellograph, demonstrate its performance on three published scRNA-seq datasets, and benchmark it against previously published methods using cross-categorical entropy and normalized mutual information [17].

## Methods

### Overview of the Cellograph algorithm

Cellograph uses GCNs to perform node classification on cells from multiple samples to quantify how representative cells are of each sample. We found GCNs to be most apropos for our implementation as they explicitly draw upon neighborhood information to capture transcriptomic relationships between cells by considering the connections between neighboring cells (e.g., molecularly similar cells) in the graph. Furthermore, as scRNA-seq is prone to technical artifacts, such as dropouts or noise in gene expression measurements, GCNs can mitigate the impact of this noise by leveraging the collective information from neighboring cells in the graph as it maps the initial dataset to a latent embedding in the first layer. By propagating information through the graph structure, GCNs can capture more reliable and robust representations of cells, improving common downstream analysis tasks like clustering, dimensionality reduction, and classification, as we shall demonstrate in the results section. Finally, the GCNs offer interpretability by learning feature importance within the context of the graph structure. By examining the learned weights in the GCN layers, we can identify features (or genes, in this context) that contribute significantly to the model’s predictions. This facilitates identification of biologically meaningful genes that drive cellular tendencies towards one experimental group versus another. This information can complement and corroborate findings from differential gene expression, but with an emphasis on group truth labels versus independently-inferred clusters.

**Fig. 1:**
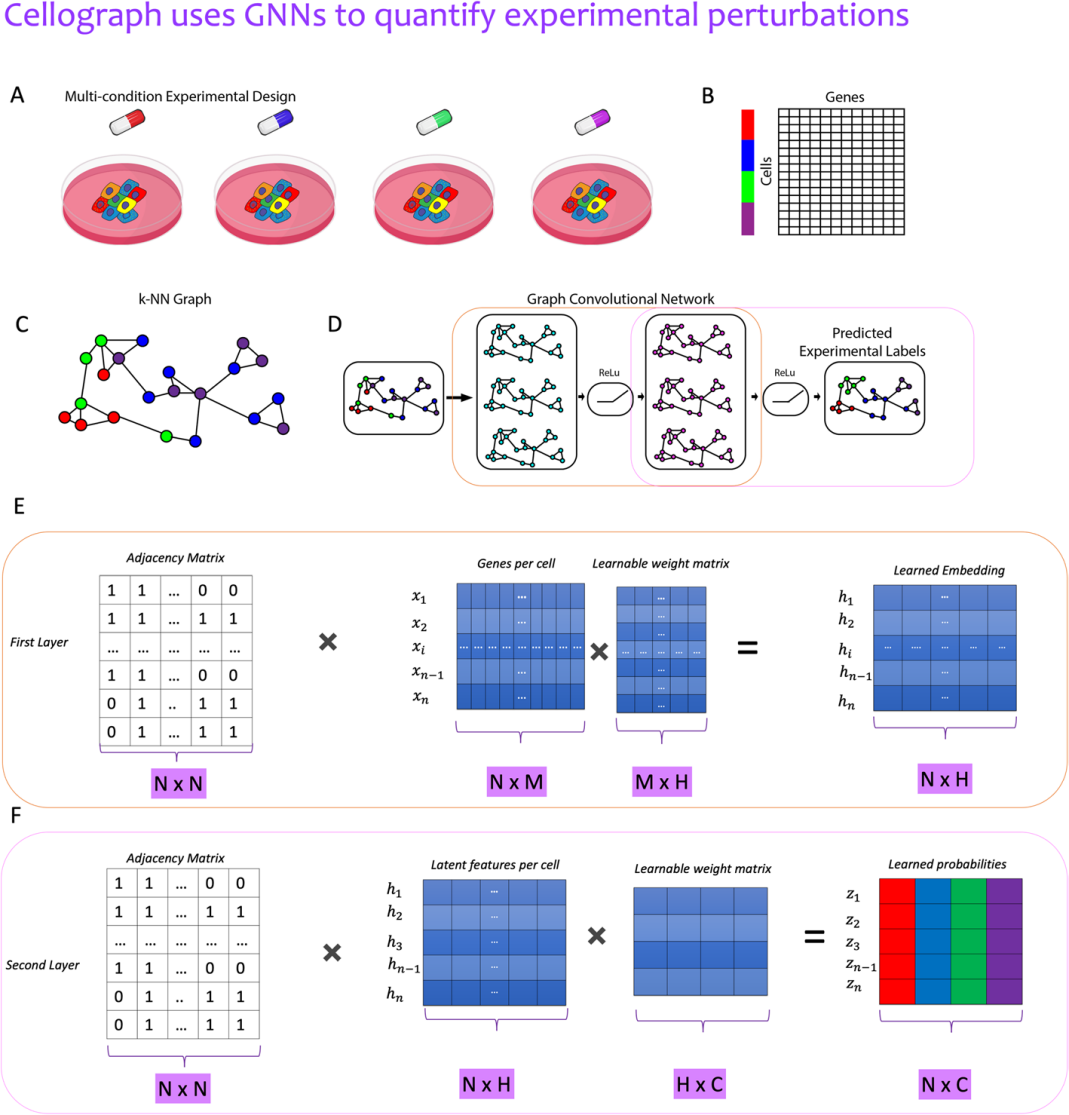
Illustrative overview of Cellograph algorithm. Single-cell data collected from multiple sample drug treatments (**A**, **B**) is converted to a kNN graph (**C**), where cells are nodes, and edges denote connections between transcriptionally similar cells. The colored rectangles (**B**) correspond to the different samples represented by the drugs in **A**. This kNN is fed in as input to a two-layer GCN (**D**) that quantitatively and visually learns how prototypical each cell is of its experimental label through the learned latent embedding. **E** A mathematical schematic of the first layer, where each cell’s gene expression and its neighbors’s gene expression is aggregated to produce a lower-dimensional representation of the cell in a latent space. **F** A mathematical schematic of the second layer respectively, where the output embedding of the first layer is mapped to softmax probabilities of cells belonging to each of the drug treatments

In summary, Cellograph takes in a single-cell dataset $X \in \mathbb {R}^{n \times m}$ (where *n* denotes the number of cells or nodes, while *m* represents the number of genes or features) aggregated from multiple treatment conditions. We assume *X* has already been pre-processed and filtered according to typical pre-processing steps when working with scRNA-seq data. (see Fig. 1). *X* is then reduced to a PCA space, where a *k*-nearest neighbor graph is constructed using a select number of principal components (PCs), with a resulting adjacency matrix $A \in \mathbb {R}^{n \times n}$. The graph is then passed in as input into a two-layer graph neural network that uses a parameterized matrix weighed by the genes to encode each cell’s transcriptome to lower dimensions that take into account the connectivity between cells. Specifically, we train a two-layer GCN on this derived graph.

In the first layer (Fig. 1D), we perform the following mathematical operation:

$$\begin{aligned} H^{(0)}=\sigma ({\hat{D}}^{-\frac{1}{2}}{\hat{A}}{\hat{D}}^{-\frac{1}{2}}XW^{(0)}). \end{aligned}$$

Here, ${\tilde{A}}$ is calculated as ${\hat{D}}^{-\frac{1}{2}}{\hat{A}}{\hat{D}}^{-\frac{1}{2}}$, and ${\hat{A}}$ is obtained by adding the identity matrix *I* to the adjacency matrix *A*. This adds self-loops to the adjacency matrix such that each cell is incorporating its own features in addition to its neighbors. $W^{(0)} \in \mathbb {R}^{m \times h}$ is a parameterized weight matrix that’s updated throughout the training of the model. Each row of the matrix corresponds to a gene, with a set of *h* weights per gene. Upon successful training, these weights can be summed up per row and ordered from highest to lowest, where genes with the highest weights denote biologically meaningful genes that distinguish the ground truth conditions. In other words, genes that more effectively distinguish conditions are given higher weights during the training. $H^{(0)} \in \mathbb {R}^{n \times h}$ is the output of the first layer in *h* latent dimensions. This matrix can be further reduced to 2 dimensions for visualization using a dimensionality reduction method like PHATE or UMAP [18]. This additional pre-processing step prior to visualization creates an embedding where cells are arranged not just according to transcriptomic similarity, but also how representative they are of each experimental condition. In the second and final layer, we have a very similar operation

$$\begin{aligned} H^{(1)}=\text {softmax}({\hat{D}}^{-\frac{1}{2}}{\hat{A}}{\hat{D}}^{-\frac{1}{2}}H^{(0)}W^{(1)}), \end{aligned}$$

where now $W^{(1)} \in \mathbb {R}^{h \times c}$ with *c* as the number of conditions. Here, we take our latent embedding $H^{(0)}$ from the initial layer and apply the same operation, only this time we map it to a matrix of treatment probabilities for each cell, giving a single-cell metric of how responsive the cell is to each treatment. The output $H^{1} \in \mathbb {R}^{n \times c}$ is a matrix of treatment probabilities. The softmax function is a nonlinear function that converts its inputs to a probability distribution proportional to the exponentials of the inputs as follows:

$$\begin{aligned} \text {softmax}({\hat{z}}) = \frac{e^{z_{i}}}{\sum _{j=1}^{K}e^{z_{j}}}. \end{aligned}$$

Regarding the training process, as noted, GNNs learn in a semi-supervised manner. This means that during training, the entire graph is observed, but only a fraction of the nodes have labeled information. Specifically, we randomly select 1–3$\%$ of nodes from each condition as training nodes, while a larger fraction are held out for testing and validation. This random selection of nodes facilitates objective training. The quality of the training is assessed via a categorical cross-entropy loss function. By default, we train the GNN for 200 epochs and terminate training if there is no improvement after 30 epochs (patience). This is in contrast to MELD, which uses all the labels and is not holding out anything, leveraging the full cell-type information via these ground-truth labels to perform the calculations, instead.

### Pre-processing the scRNA-seq data

We pre-process data as commonly done using Scanpy, unless specified otherwise [19, 20]. For the organoid dataset, we downloaded the publicly available, normalized dataset from https://singlecell.broadinstitute.org (study SCP1318) and filtered the most highly variable genes (using the default parameters in Scanpy: a minimum mean expression of 0.0125, maximum mean expression of 3, and minimum normalized dispersion of 0.5). Metadata was also included with cell type annotation and ground truth treatment groups. For the drug holiday dataset, we followed the pre-processing steps described in the original study, only implementing them in Scanpy over Seurat. For the myogenesis dataset, we followed the quality control steps described in the original paper, except implemented in Scanpy rather than Seurat (all cells with less than 300 genes expressed were removed, as well as all genes expressed in less than 10 cells; furthermore, only cells with less than 20$\%$ percentage mitochondria expression were retained). The data was then normalized using Scanpy to 10,000 reads per cell, logarithmized, and filtered down to the top 2000 highly variable genes.

## Results

We demonstrated the biological application of Cellograph on three published scRNA-seq datasets: a human organoid model of intestinal stem cells differentiating to Paneth cells with or without a stimulus to enhance the efficiency of the differentiation [21]; a non-small-cell lung carcinoma (NSCLC) cell line that was treated with a drug called Erlotinib at various time points and later temporarily withdrawn from the drug for several days [22]; and a myogenesis model of transdifferentiation and traditional cell reprogramming [23]. We benchmarked the performance of Cellograph against the aforementioned differential abundance methods, MELD, Milo, and CNA. Our results show robust performance of Cellograph on these distinct datasets, and provide valuable biological insights.

### Cellograph captures shifts in cell type abundance during human intestinal organoid differentiation

**Fig. 2:**
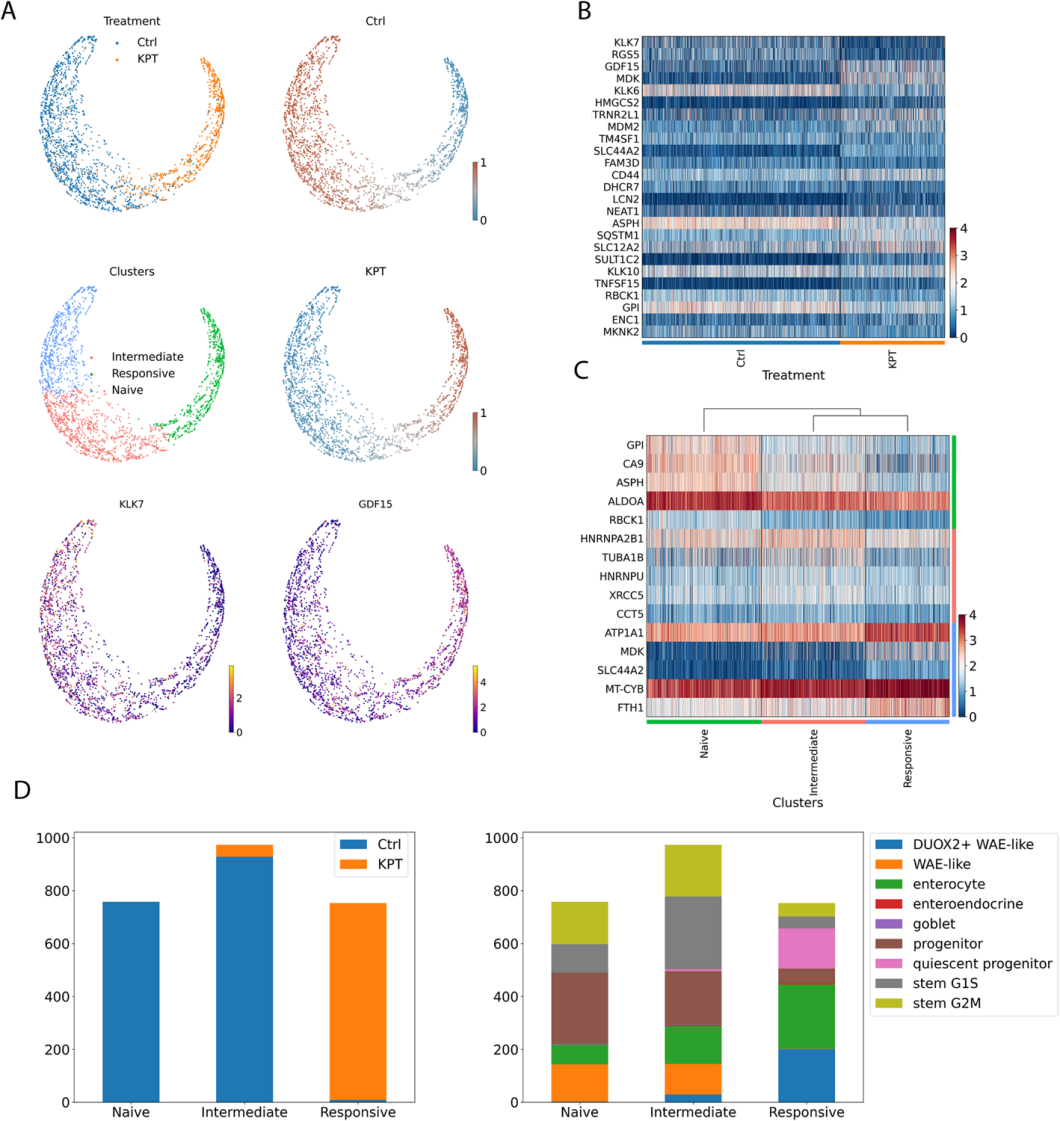
Cellograph identifies treatment groups and distinguishes genes defining these groups on a human organoid dataset. **A** PHATE projection of learned latent space, with cells colored by treatment labels, probabilities of belonging to control or KPT-treated cells, clusters obtained by k-means clustering of the learned latent embedding with $k=3$, and gene expression of GDF15 and KLK7. **B** Heatmap of top 25 weighted genes from parameterized gene weight matrix. **C** Heatmap of differentially expressed genes between clusters derived from Cellograph. **D** Compositional plot of predicted treatment groups from the softmax probabilities ($z_{ij} > 0.5$) (left) and cell types annotated by the original study (right) partitioned by clusters

We first applied Cellograph to an organoid model of intestinal stem cells differentiating to Paneth cells with or without KPT-330, an inhibitor of the nuclear exporter, Exportin 1, which was demonstrated in the original study [21] to enhance the abundance of Paneth cells following differentiation. Samples were collected from 6 donors for sequencing following 6 days of treatment with or without KPT-330. Cell type annotation revealed 9 prominent cell types: Stem cells transitioning from G1 to S phase of the cell cycle (G1/S), stem cells in G2 and M phase of the cell cycle, proliferative progenitor cells (Progenitor), enterocytes (Enterocyte), wound-associated epithelium cells (WAE), WAEs enriched in the well-characterized stress-associated gene DUOX2 (DUOX2+ WAE-like), quiescent progenitors (Quiescent progenitor), goblet cells (Goblet), and enteroendocrine cells (Enteroendocrine). We will refer to these two conditions as KPT and control cells, respectively. We trained Cellograph using a two-layer GCN with 80 out of the 2484 cells labeled, such that 40 were labeled for each condition. We projected the learned latent space to 2 dimensions with PHATE and colored cells according to the probability of belonging to the KPT-treated condition (Fig. 2A). We obtain a smooth gradient of cells along the PHATE plot, with cells arranged according to how impacted they are by KPT treatment. UMAP also captured the separation between conditions and gradient of probability scores [18] compared to traditional UMAPs on the high-dimensional PCA space (Additional file 1). To determine if this gradient reflected meaningful biology, we extracted the 25 top-weighted genes from the aforementioned learned weight matrix (discussed in “Overview of the Cellograph algorithm” section) and visualized them with a heatmap categorized by the two treatment groups (Fig. 2B), which corroborates existing findings for the source paper. This matrix is derived from the first layer of the GCN and parameterizes each gene, where the model upweights genes it finds most relevant in distinguishing between conditions. Among the top 25 genes is GDF15, a marker of DUOX2+ WAE-like and WAE-like cells, which is highly expressed in KPT-treated cells, where these cell types are more abundant due to the greater efficiency of Paneth cell differentiation [24, 25]. Conversely, KLK6 is highly expressed in the control-treated population, which has been shown to mediate the multipotency of intestinal stem cells [26, 27].

We also performed *k*-means clustering on the latent space learned by Cellograph with $k=3$ (Fig. 2A,C). Unlike clustering the original PCA-reduced data, which just focuses on differences in the transcriptome, Cellograph implicitly clusters according to how responsive cells are to the KPT-330 stimulus. This successfully groups together cells predicted to belong to the KPT-treated group (called the responsive cluster), a mixed population of cells predicted to be either control or KPT-treated cells (intermediate cluster), and a cluster of cells predicted to be prototypical of the control population (naive cluster). These predictions were determined using a threshold of 0.5 for ground truth assignment.

Based on the softmax probabilities learned by Cellograph ($z_{i,j} \ge 0.5$), we assigned cells to the control or KPT-treated populations independent of their ground truth labels, and created composition plots according to cluster assignment (Fig. 2D). We see that Cellograph’s predictions corroborate the compositional changes in cell types abundance discussed in the original study, namely with decreases in dividing stem cell and progenitor populations, increases in quiescent progenitors, enterocytes, and DUOX2+ WAE-like cells.

Finally, we mapped the cell type annotations onto the clusters obtained by Cellograph (Fig. 2D) and observe a high abundance of cycling cells, progenitor cells, and WAE-like cells in the Naive cluster, followed by a decrease of WAE-like cells and progenitor cells in the intermediate population, and a high proportion of DUOX2+ WAE-like cells in the responsive cluster. Altogether, these results demonstrate Cellograph’s ability to identify and visualize cells affected by KPT-330 stimulation. It corroborates existing findings and presents an interpretable framework for downstream tasks like visualizing and clustering the data.

### Cellograph models heterogeneity in cancer drug response during a drug holiday

**Fig. 3:**
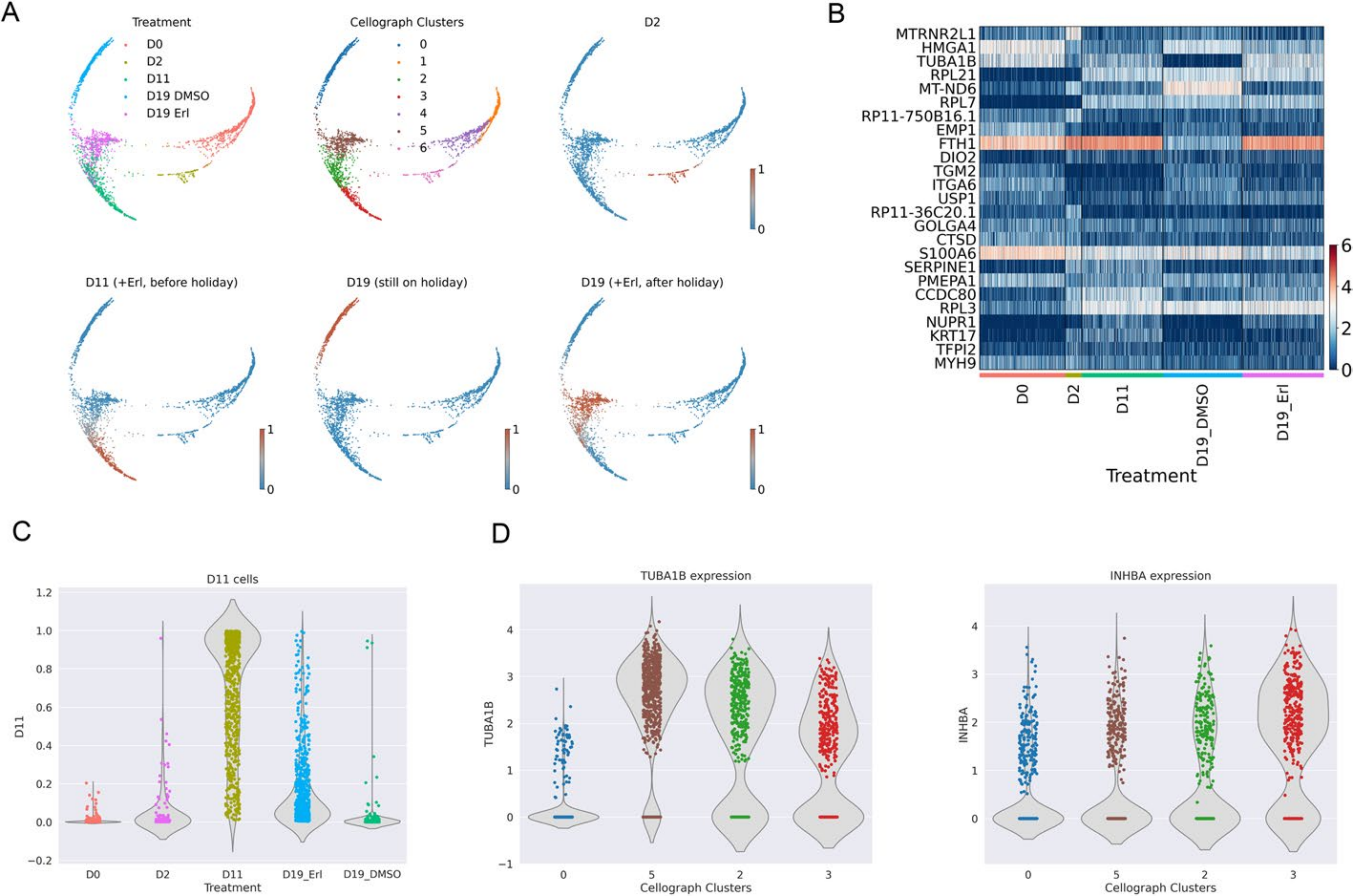
Cellograph defines genetic signatures of distinct drug responses in the drug holiday dataset. **A** PHATE embeddings of the learned latent space colored according to the treatment labels, clusters, and treatment probabilities (day 0 not shown). **B** Heatmap of top 25 weighted genes from learned parameterized gene weight matrix. **C** The distribution of treatment probabilities for Day 11 cells partitioned by treatment groups. **D** The distribution of gene expression between clusters 0, 5, 3, and 2 of select differentially expressed genes (INHBA, TUBA1B)

Encouraged by Cellograph’s performance on the human intestinal organoid dataset, we next investigated how well it could capture heterogeneity in response to cancer drugs under complex treatment regimes. We trained Cellograph on the single-cell transcriptomes of 3042 PC9 cells treated with Erlotinib [22]—a tyrosine kinase inhibitor used to treat non-small cell lung cancer (NSCLC)—for 11 days, followed by withdrawal of the drug for 6 days, referred to as a drug holiday, where select cells were either retreated with Erlotinib or treated with DMSO as a control. This study examined the drug-tolerant states in a non-small-cell lung carcinoma (NSCLC) cell line, where the goal was to understand what cell populations would emerge from treatment and retreatment. Specifically, the authors treated the cell line with a drug called Erlotinib for 11 days, followed by a 6-day withdrawal period called a drug holiday as the cells developed resistance. A subset of cells was then reintroduced to Erlotinib for 2 days and cells were sequenced at each time point. The key takeaway from this paper was that they identified subpopulations of cells associated with genes that induced drug resistance, and those inhibiting drug resistance. However, this just considered transcriptomic variation and simple graph-based Leiden clustering, so we were interested if Cellograph could quantify the effect of these temporal perturbations at single-cell resolution, corroborate these findings, and perhaps offer novel insights into these mechanisms of drug resistance. The cells were sequenced at 5 timepoints: 0 days with no Erlotinib treatment, 2 days of Erlotinib treatment, 11 days of Erlotinib treatment, at day 19 with or without re-exposure to Erlotinib on day 17, following 6 days of removal from the drug. We trained Cellograph on these PC9 cells with 30 cells labeled for each condition using a 2-layer GCN. We project the learned latent space into 2 dimensions with PHATE, which gives a clear temporal separation of the 6 treatment groups (Fig. 3A), comparable to UMAP (Additional file 1). Coloring cells according to the probability of belonging to each of the conditions provides a narrow distribution of scores in cells in the condition of interest, with the notable exception of Day 11 (Erlotinib before holiday) and Day 19 (Erlotinib after holiday), suggesting a non-uniform response to the drug in these cells both before and after the drug holiday (Fig. 3D). The heatmap of the top 25 weighted genes from training (Fig. 3B) implicates such genes as TUBA1B and CCDC80 in distinguishing the conditions, which are both markers of drug resistance, with CCDC80 highly expressed in D11 cells, corroborating the original study’s observations of CCDC80, whereas TUBA1B expression is particularly elevated in D19 Erl cells. Almost all of these genes were previously identified through differential gene expression in the original paper, showcasing Cellograph’s interpretability of the weigh matrix in identifying pertinent genes defining molecular differences. However, MT-ND6, which was not among the differentially expressed genes to the best of our knowledge, is also strongly weighted and appears to uniformly define the population of cells that were treated with DMSO following the drug holiday. This is a mitochondrial gene which has been previously implicated in colorectal adenocarcinoma and associated with changing energy requirements due to cells aggressively proliferating [28]. Clustering the learned latent space identifies three clusters among these two conditions (Fig. 3A,D), one consisting of cells predicted to have a prototypical response after 11 days of Erlotinib treatment (cluster 3), and similarly for day 19 after re-exposure to the drug (cluster 5), followed by a mixed population of both cell types (cluster 2). Differential expression between the three clusters (Fig. 3C) identified high expression of TUBA1B in cluster 5, which is associated with poor prognosis in NSCLC, suggesting persisting drug tolerance after the holiday period. Similarly, we observe differential expression of INHBA in cluster 3, a senescence mediator that’s associated with prognosis in many cancer types [29]. This suggests that there is drug resistance in both treatment regimes, yet seemingly stimulating different pathways of resistance as opposed to anti-resistance, highlighting the limitations of the treatment scheme. Interestingly, TUBA1B and INHBA expression are significantly reduced in the day 19 population that was not retreated with Erlotinib. Altogether, Cellograph captures clinically relevant genes driving heterogeneity in response to treatment, corroborates existing findings of pertinent genes driving treatment response, identifies an additional gene that was previously not described to the best of our knowledge, and suggests different modes of drug resistance.

### Cellograph distinguishes between transdifferentiation and dedifferentiation in myogenesis

Finally, we assessed Cellograph’s ability to distinguish cells undergoing distinct cell state transitions temporally on a scRNA-seq dataset of 33,380 mouse embryonic fibroblasts (MEF) undergoing either dedifferentiation to adult stem cells called induced myogeneic progenitor cells (iMPCs) or myogenic transdifferentiation to myotubes [23]. The original study was motivated to understand the transcriptional and epigenetic mechanisms of how over-expression of the MyoD transcription factor induced MEFs to undergo reprogramming to either myotubes or iMPSCs with a MyoD-inducible transgenic model. The myotubes were induced by overexpression of MyoD, while the addition of small molecules produced $\text {Pax7}^{+}$ iMPSCs that were very similar to primary muscle stem cells. The authors used trajectory analysis via diffusion maps and UMAP embeddings of combined single-cell data of MEFs expressing MyoD or MyoD + a cocktail of small molecule inhibitors (forskolin, RepSox, and CHIR99021, collectively abbreviated as “FRC” in the original paper) to reveal that dedifferentiation and transdifferentiation follow two different trajectories.

**Fig. 4:**
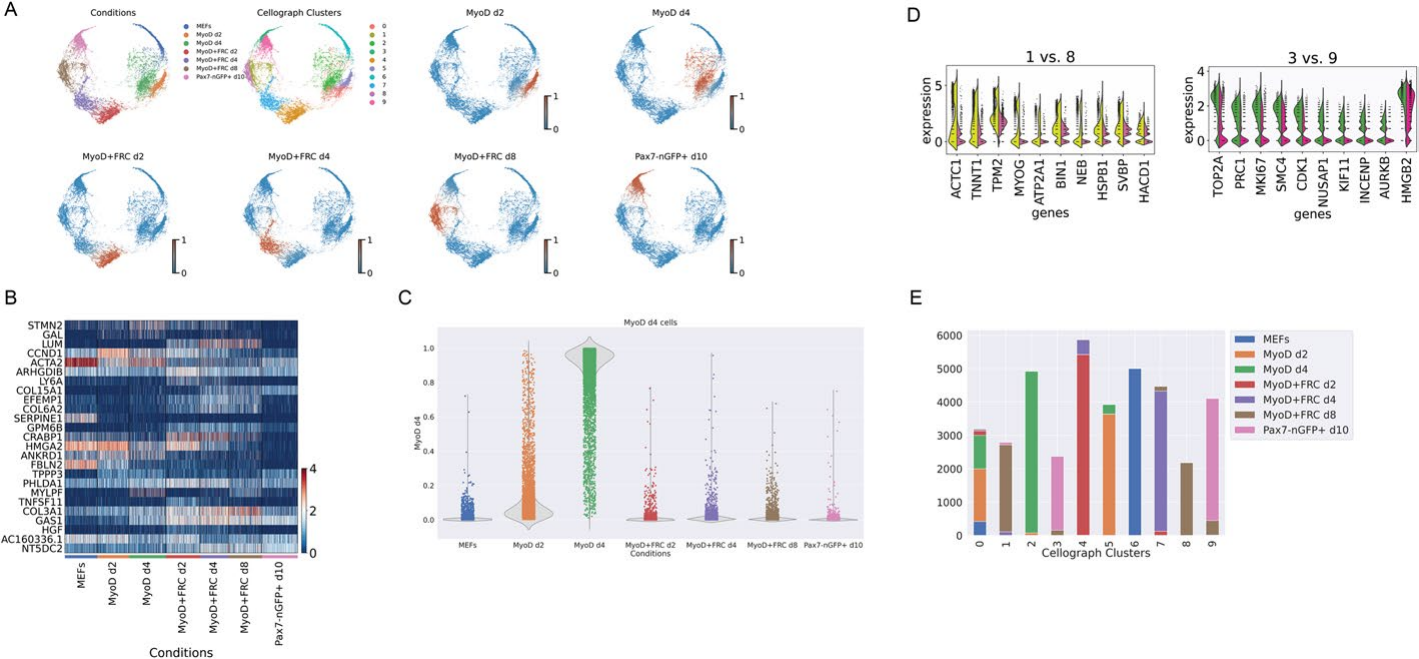
Cellograph distinguishes the molecular mechanisms of transdifferentiation and dedifferentiation in myogenesis. **A** PHATE embeddings of learned latent space annotated according to treatment conditions, clusters, and softmax probabilities of all conditions except for MEFs, defining the in-group variation. **B** Heatmap of top weighted genes from parameterized gene weight matrix, identifying pertinent genes such as cyclin D1 and CRABP1. **C** Violin plot of softmax probabilities of cells belonging to the MyoD/day 4 treatment group, showing similarities to the MyoD/day 2 population. **D** Violin plots of top 20 differentially expressed genes between clusters 1 and 8 and clusters 3 and 9, which define the $\text {Pax7}^{+}$ cells and MyoD+FRC/day 8 treated cells, respectively. **E** Compositional plot of predicted cell types partitioned by cluster

We trained Cellograph on these differentiating cells with 200 cells per treatment group labeled for training for 400 epochs and obtained a single trajectory that starts with transdifferentiation and culminates in dedeifferentiation to $\text {Pax7}^{+}$ iMPCS (Fig. 4A; Additional file 1). Looking at the top-weighted genes from training the model (Fig. 4B), high expression of CRABP1 and LUM distinguished the transdifferentiating population, whereas dedifferentiation was weighted by high expression of cyclin D1, suggesting cell cycle entry is a necessary step to producing iMPCs. CRABP1 is known to promote stem cell proliferation by its downregulation [30]. However, it does not appear to inversely vary with cyclin D1. The original study revealed an overlap between the major fraction of day 4 MyoD-treated cells and day4/8 MyoD+FRC-treated cells in their UMAP and DPT embeddings. Interestingly, however, Cellograph detects no significant overlap (Fig. 4A), which is further supported by the derived probabilities of belonging to each of the experimental groups (Fig. 4C).

STMN2, an early neuronal marker, was also identified as a pertinent gene in distinguishing between these processes (Fig. 4B), with high expression in the transdifferentiation condition, perhaps owing to the instability and inefficiency of generating myotubes with MyoD alone. Clustering the latent space and mapping the clusters onto the PHATE embedding distinguished the different treatment conditions and heterogeneity in the MyoD+FRC day 8 condition. Notably, we observed differential expression of MYOG (Fig. 4D), which specifies the myotube fate, in the majority of cells, which corroborates observations from trajectory analysis in the original study where this gene is observed in both trajectories. Cell cycle differences underscored variability in the $\text {Pax7}^{+}$ iMPCs (Fig. 4D,E). Altogether, Cellograph is able to successfully distinguish these biological processes, and identify additional gene programs explaining these differences.

### Cellograph outperforms published differential abundance methods and popular single-cell clustering methods

Finally, we benchmarked Cellograph’s performance in identifying cells most impacted by perturbations against three MELD, Milo, and CNA. We used the Brier score for comparison between Cellograph and MELD as we believed a method quantifying experimental perturbations should capture a broad range of signals for each experimental label it is trying to predict. In particular, this metric quantifies the squared difference between predicted and true probabilities distributions by calculating the following sum,

$$\begin{aligned} \text {BrierScore}(y, p) = \frac{1}{N} \sum _{i=1}^{N} \sum _{j=1}^{C} (p_{ij} - \delta _{ij})^2 \end{aligned}$$

where *y* represents the true labels of the samples, with $y_i \in \{1, 2, \ldots , C\}$ denoting the true class label of sample *i*, *p* represents the predicted probabilities of the samples, with $p_{ij}$ denoting the predicted probability of sample *i* belonging to class *j*, *N* represents the total number of samples, and $\delta _{ij}$ is the Kronecker delta function defined as $\delta _{ij} = 1$ if $y_i = j$ and $\delta _{ij} = 0$ otherwise. Lower values reflect better quality performance. When applied to all the cells in our datasets, we obtain consistently lower scores than MELD (Table 1), despite MELD using all class labels during its learning process whereas Cellograph uses only a fraction.

**Fig. 5:**
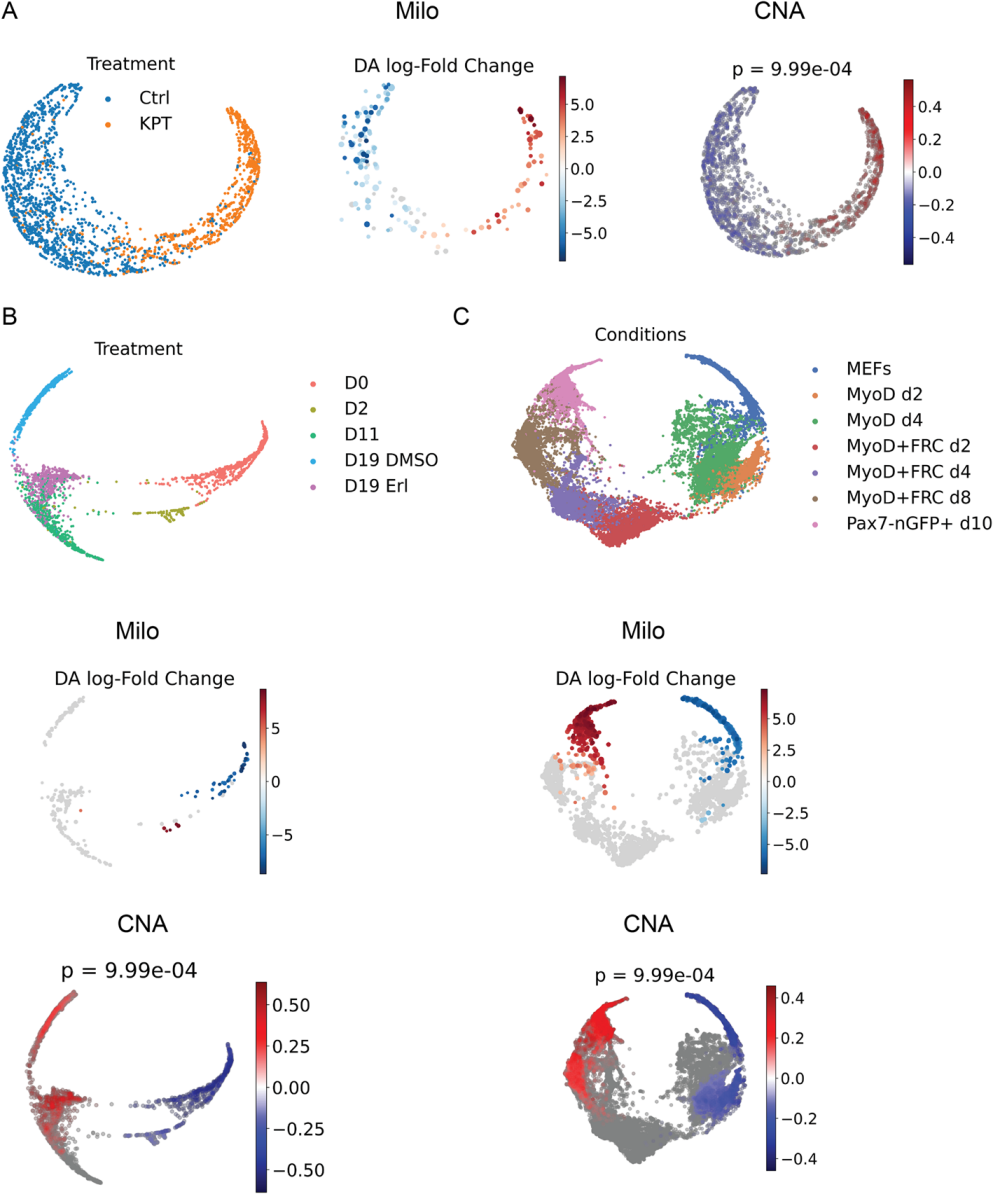
Results of running Milo and CNA on the datasets evaluated. **A** Output of running Milo and CNA on the human organoid dataset. **B** Output of running Milo and CNA on the drug holiday dataset. **C** Output of running Milo and CNA on the myogenesis dataset

When evaluating Cellograph relative to Milo and CNA, however, we could not perform direct quantitative comparison. As discussed in “Related methods” section, Milo gauges the presence of differential abundance on kNN graphs by aggregating cells into overlapping neighborhoods and performing a quasi-likelihood F test. This returns a metric of the log-fold change of the differential abundance in each neighbor, not a single-cell measurement giving the probability of that cell belonging to one treatment class versus another. Thus, we cannot perform a direct quantitative comparison and instead present a qualitative assessment of performance. Running Milo on the human organoid dataset, we observe a positive correlation between the Milo-derived log-fold changes in differential abundance and the probability of cells belonging to the KPT-treated group (Fig. 5A). However, when applied to the the drug holiday and myogenesis datasets (Fig. 5B,C), which have more complex experimental designs with multiple conditions, Milo fails to yield clear, interpretable results, with low DA in the untreated population, high DA in the cells after one day of Erlotinib treatment, and minimal DA in all other conditions. Similarly, in the myogenesis dataset, we observe high DA in Pax7-treated cells, low DA in MEFs, and minimal DA everywhere else.

**Fig. 6:**
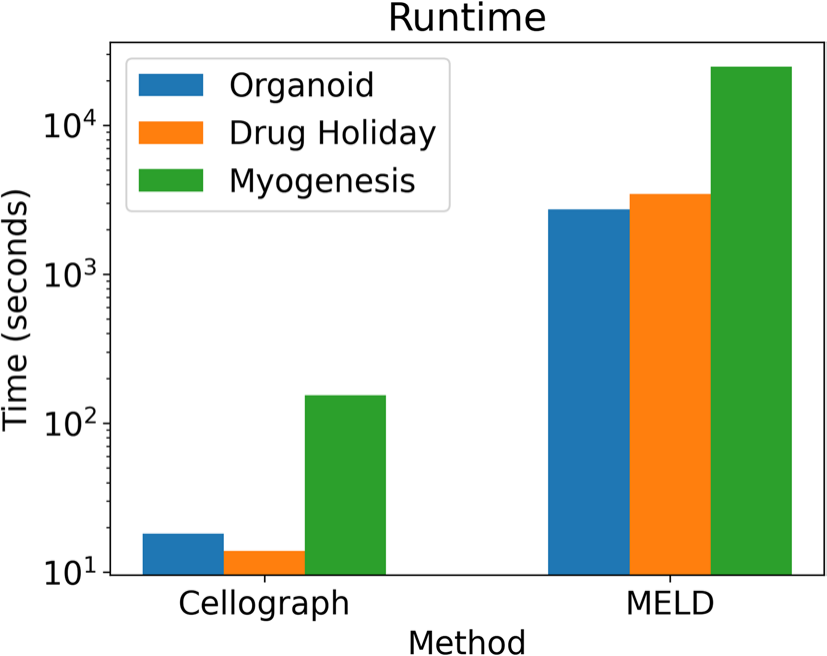
Runtime of Cellograph’s performance versus MELD’s on optimal parameter settings. Cellograph consistently outperforms MELD on each dataset, while using fewer computing resources (y-axis is log-scaled)

Applying CNA to the human organoid dataset with the KPT treatment status as the attribute of interest, we obtain similar results as our method, MELD, and Milo. Specifically, we observe high correlation in the KPT-treated cells, and low correlation in the untreated cells. This elevated correlation is on par with the high probability of observing cells in the KPT-treated group. However, on the Erlotinib and myogenesis datasets, like Milo, we obtain results incongruous with Cellograph or MELD’s performance. It is even at odds with Milo. High abundance is predicted for cells treated right before holiday and following the holiday, regardless of whether cells were retreated with Erlotinib, whereas low abundance is observed in both the untreated cells and cells treated with Erlotinib for one day, while cells with 11 days of treatment have zero correlation. Since this dataset spans multiple conditions and CNA just calculates one set of metrics, it was difficult to interpret these results in the context of the experiment. We obtained similarly incongruous results for the myogenesis dataset (Fig. 5). Altogether, Cellograph provides robust and interpretable results for more complex experimental designs with multiple treatment groups compared to CNA and Milo, and performs consistently better than MELD with a significantly lower runtime for optimal performance (Fig. 6).

**Fig. 7:**
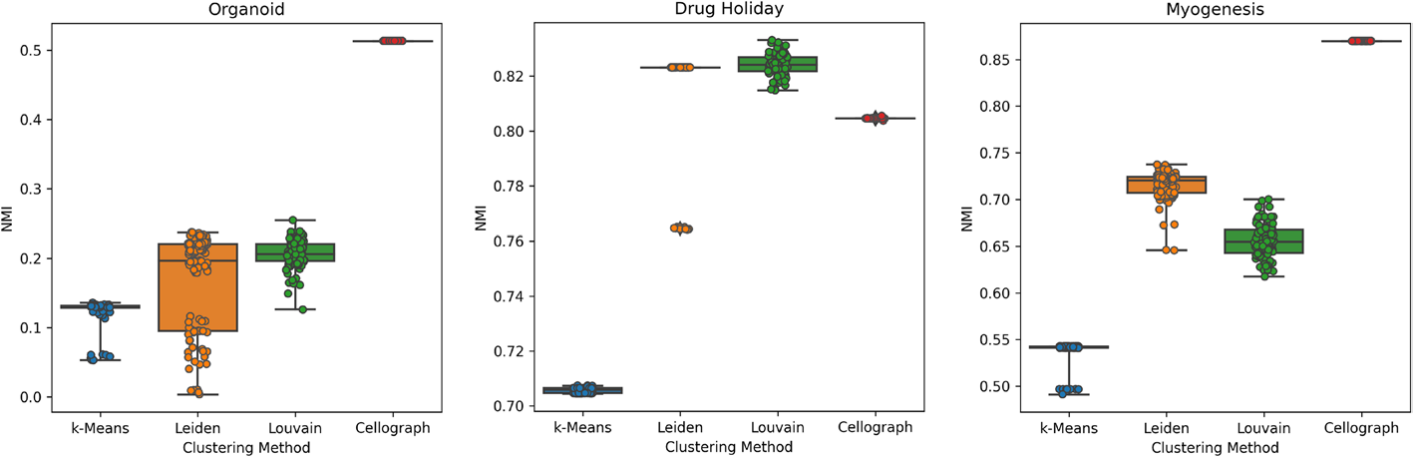
Boxplots of NMI values per clustering algorithm. Distributions of 100 independent NMI calculations for each clustering algorithm for all three datasets evaluated, quantifying concordance between the cluster assignments and ground truth labels

When evaluating clustering performance with NMI, *k*-means clustering on the learned latent space yielded consistently high metrics compared to the Leiden and Louvain clustering algorithms, and *k*-means clustering on data in PCA space. 100 NMI values were calculated for each dataset by performing independent runs of the clustering algorithms. (Fig. 7). The treatment annotations given in the source papers were used as ground truth to derive the NMI values. Resolution parameters for the most optimal number of clusters in the Leiden and Louvain were chosen such based on the scib software [31] for more rigorous comparison (Table 2).

Resolution parameters were chosen such that the Leiden and Louvain algorithms generated the same number of clusters as *k* for *k*-means clustering for a more rigorous comparison (for the organoid dataset, resolution parameters of 0.3 and 0.2 were chosen for Louvain and Leiden clustering, respectively; for the drug holiday dataset, resolution parameters of 0.6 and 0.45 were chosen for Louvain and Leiden clustering, respectively; and for the myogenesis dataset, resolution parameters of 0.45 and 0.34 were chosen for Louvain and Leiden clustering, respectively).

However, we stress that this improvement in clustering is not a novel contribution of Cellograph. Ultimately, we are still performing *k*-means clustering, however, the input to the simple clustering algorithm is what impacts the performance. Traditional clustering methods like *k*-means perform clustering on a lower-dimensional PCA representation of the single-cell data. However, instead of a linear transformation of the data, we perform a non-linear transformation prior to clustering via the initial layer of the GCN. The clustering method itself is not novel, but the way the data is processed prior to clustering is. Instead, we emphasize that the novel contribution of Cellograph is a scalable means of ascertaining the effects of different experimental regimes at single-cell resolution.

Examining the sensitivity of the hyperparameters (the number of neighbors *k*, the latent dimension *h*, and principal components PCs) during our training by looking at the learning curves of accuracy and loss, we found Cellograph had fairly consistent performances with a minimum of $h=16$ latent dimensions across all 3 datasets (Additional files 2, 3, and 4, for organoid, drug holiday, and myogenesis, respectively for tables of validation accuracy metrics for each combination of parameters).

Altogether, Cellograph outperforms MELD is estimating how prototypical cells are of their ground truth labels, and consistently ranks higher than standard algorithms for clustering.

**Table 1:** Brier score of Cellograph versus MELD on all cells

Dataset	Cellograph	MELD (optimal settings)
Organoid	0.153	0.362
Drug holiday	0.133	0.222
Myogenesis	0.094	0.324

**Table 2:** Average NMI score from different clustering methods on the datasets (standard deviation of scores in parentheses) along with optimal resolution parameter or choice of *k* used in each method

Dataset	K-means on PCA space	Leiden	Louvain	K-means on Cellograph-derived latent space
Organoid	$k=8$ 0.124 (0.019)	Resolution = 0.2 0.183 (0.071)	Resolution = 0.4 0.183 (0.021)	$k=2$ 0.513 (6.695 $\times 10^{-16}$)
Drug holiday	$k=4$ 0.706 (9.255$\times 10^{-4}$)	Resolution = 0.2 0.817 (0.018)	Resolution = 0.2 0.824 (3.956$\times 10^{-3}$)	$k=5$ 0.805 (1.292$\times 10^{-4}$)
Myogenesis	$k=10$ 0.538 (0.013)	Resolution = 0.1 0.715 (0.016)	Resolution = 0.5 0.655 (0.018)	$k=7$ 0.87 (5.174$\times 10^{-5}$)

## Discussion

When designing single-cell experiments exploring the impacts of different treatments, it is vital to leverage the heterogeneity present at such resolution. The increasing complexity of the experimental design (e.g., multiple treatments, various timepoints, etc) can result in diminishing returns from standard differential gene expression and clustering approaches due to the biological and technical variability present at the single-cell level. Existing approaches like MELD and VFC are apt for studying the effects of one experimental treatment, but cannot be easily generalized to more complicated experimental programs. We designed Cellograph to address this challenge. Beyond just quantifying single-cell responses to perturbations analogously to MELD, Cellograph’s primary innovation lies in its novel way of visualizing and clustering single-cell data by means of graph neural networks, which, through the parameterized gene weight matrix, provides an interpretable means of understanding which genes drive the difference between conditions. We have shown that our approach improves clustering on three diverse datasets compared to standard clustering approaches, as well as captures a stronger signal of the ground truth experimental label compared to MELD. Clustering agnostic of experimental conditions can fail to take into consideration the diversity of cellular responses to these perturbations and how those correspond to the transcriptomic variation. By applying simple k-means clustering to the latent space, we can obtain more informative clusters that enable deeper biological insight, especially in populations under the same experimental treatment. In addition to improved differential gene expression, we also obtain complementary information from the parameterized weight matrix after training, which reveals the most important genes in distinguishing between different treatments.

In a published dataset of donor-provided organoid samples, we were able to successfully corroborate original findings, while providing a visually informative view of the data, and revealed novel insights into drivers of KPT-mediated organoid differentation. Similarly, in our drug holiday application, we identified additional markers of drug resistance using the parameterized gene weight matrix, and described heterogeneity of cells in response to Erlotinib after 11 days and post-holiday, while characterizing the popular that was retreated after the holiday that could inform future experiments into druggable targts for NSCLC. Finally, in our myogenesis evaluation, we identified shared features between transdifferentiation and dedifferentiation, while capturing relevant markers that distinguished the two processes. We anticipate Cellograph will find a wide range of application to other biological contexts and different single-cell modalities as an all-in-one framework for facilitating visualization, clustering, and single-cell responses to perturbations, on top of its efficiency. For example, this work could find utility in clinical applications to studying heterogeneity in patient-treated samples in response to an experimental cancer drug. This could be also employed to study impacts of cancer drugs on cell cycle in protein immunofluorescence imaging data [32]. Potential extensions of our method could certainly explore the incorporation of batch effect corrections. While this could be a valuable avenue to address potential confounding factors and improve the robustness of the analysis, we want to emphasize that the primary objective of our method, as well as other methods in similar tasks, is not specifically focused on batch effect correction, and we would advise users to independently correct for any technical artifacts prior to using Cellograph. If there are several replicates for a specific condition, the user may perform batch effect correction using approaches such as Harmony [33] or Seurat 3 [34], which have been independently shown to perform well in batch-effect correction [35].

The graph neural network architecture of node classification could even be extended to graph classification for looking at multiple patient samples, as is common in mass cytometry, or regression to predict continuous variables such as cellular pseudotime in the context of differentiation, cell cycle age [36], or gestational age in data from pregnant women [37], and may be further explored in future work. The choice of different graph constructions mechanisms could also warrant exploration in future studies. For example, CellVGAE uses variational graph autoencoders to reconstruct input graphs, adding additional, relevant edges, which can facilitate clustering and other downstream tasks in single-cell analysis [13]. scGNN is another GNN framework for single-cell analysis that selectively prunes edges in the initial *k*NN graph when pre-processing the data prior to training [9].

Concerning limitations of Cellograph, as a semi-supervised method, it requires labels to train on and make informed predictions. Consequently, in sparsely-labeled data or data with no labels at all, Cellograph’s performance may fall short. In the case of sparse data, Cellograph could be used to impute the labels of other non-annotated cells. As for unlabeled data, one could pre-train Cellograph on a cellular atlas such as the Human Lung Cell Atlas [38] and apply the trained model to the unlabeled dataset to predict different cell types or distinguish diseased cells from healthy cells. Such endeavors could be the subject of future extensions of Cellograph. Because of the aforementioned dependency on labeled data, accurate labeling is imperative in achieving meaningful interpretation of Cellograph’s results. In addition, data with multiple conditions that have similar phenotypes may present a challenge during Cellograph’s learning due to the difficulties in separating conditions in the latent space.

Altogether, Cellograph provides a novel framework for perturbation analysis, data visualization, and feature importance in single-cell genomics. We anticipate it will find utility for testing drug efficacy in clinical samples, and the incorporation of other single-cell modalities, which may be explored in future studies.

### Supplementary Information

**Additional file 1**. The additional file (SupplementalFile_cellograph.docx) provides supplementary figures of the corresponding UMAP embeddings of the respective single-cell transcriptomes on the Cellograph-derived latent space described in the main text (Figures 2, 3, and 4) and compares them to UMAPs of the multi-dimensional PCA coordinates, showcasing the visual gradient of treatment effects captured by the former visualizations.

**Additional file 2**. The additional file (S1.xlsx) provides tables examining the hyperparameter sensitivity used to evaluate Cellograph's robustness on the organoid dataset described in the main text.

**Additional file 3**. The additional file (S2.xlsx) provides tables examining the hyperparameter sensitivity used to evaluate Cellograph's robustness on the drug holiday dataset described in the main text.

**Additional file 4**. The additional file (S3.xlsx) provides tables examining the hyperparameter sensitivity used to evaluate Cellograph's robustness on the organoid dataset described in the main text.

## Data Availability

The metadata and digital gene expression data for the human organoid dataset was downloaded from https://singlecell.broadinstitute.org (study SCP1318). The Erlotinib drug holiday dataset was downloaded from the database Gene Expression Omnibus (GEO) (https://www.ncbi.nlm.nih.gov/geo) under the accession number GSE134841. The myogenesis dataset was downloaded from GEO under the accession number GSE171039. The code and installation insturctions for Cellograph can be found at https://github.com/jashahir/cellograph.
