# Supplementary material for: Cellograph: a semi-supervised approach to analyzing multi-condition single-cell RNA-sequencing data using graph neural networks
Source: BMC Bioinformatics. 2024 Jan 15;25:25. doi: 10.1186/s12859-024-05641-9 (PMC10788980; doi:10.1186/s12859-024-05641-9)
Supplement: Supplementary file 1 — Additional file 1. The additional file (SupplementalFile_cellograph.docx) provides supplementary figures of the corresponding UMAP embeddings of the respective single-cell transcriptomes on the Cellograph-derived latent space described in the main text (Figures 2, 3, and 4) and compares them to UMAPs of the multi-dimensional PCA coordinates, showcasing the visual gradient of treatment effects captured by the former visualizations. [file 12859_2024_5641_MOESM1_ESM.docx]

**Additional file 1**

**Cellograph: A Semi-supervised Approach to Analyzing Multi-condition Single-cell RNA-sequencing Data Using Graph Neural Networks**

Jamshaid A. Shahir^1,2,3^, Natalie Stanley^2,3,4^, Jeremy E. Purvis^1,2,3,5^*

1 Department of Genetics, University of North Carolina at Chapel Hill,

Chapel Hill, North Carolina, United States.

2 Curriculum in Bioinformatics and Computational Biology, University of

North Carolina at Chapel Hill, Chapel Hill, North Carolina, United

States.

3 Computational Medicine Program, University of North Carolina at

Chapel Hill, Chapel Hill, North Carolina, United States.

4 Department of Computer Science, University of North Carolina at

Chapel Hill, Chapel Hill, North Carolina, United States.

5 Lineberger Comprehensive Cancer Center, University of North Carolina

at Chapel Hill, Chapel Hill, North Carolina, United States.

*corresponding author


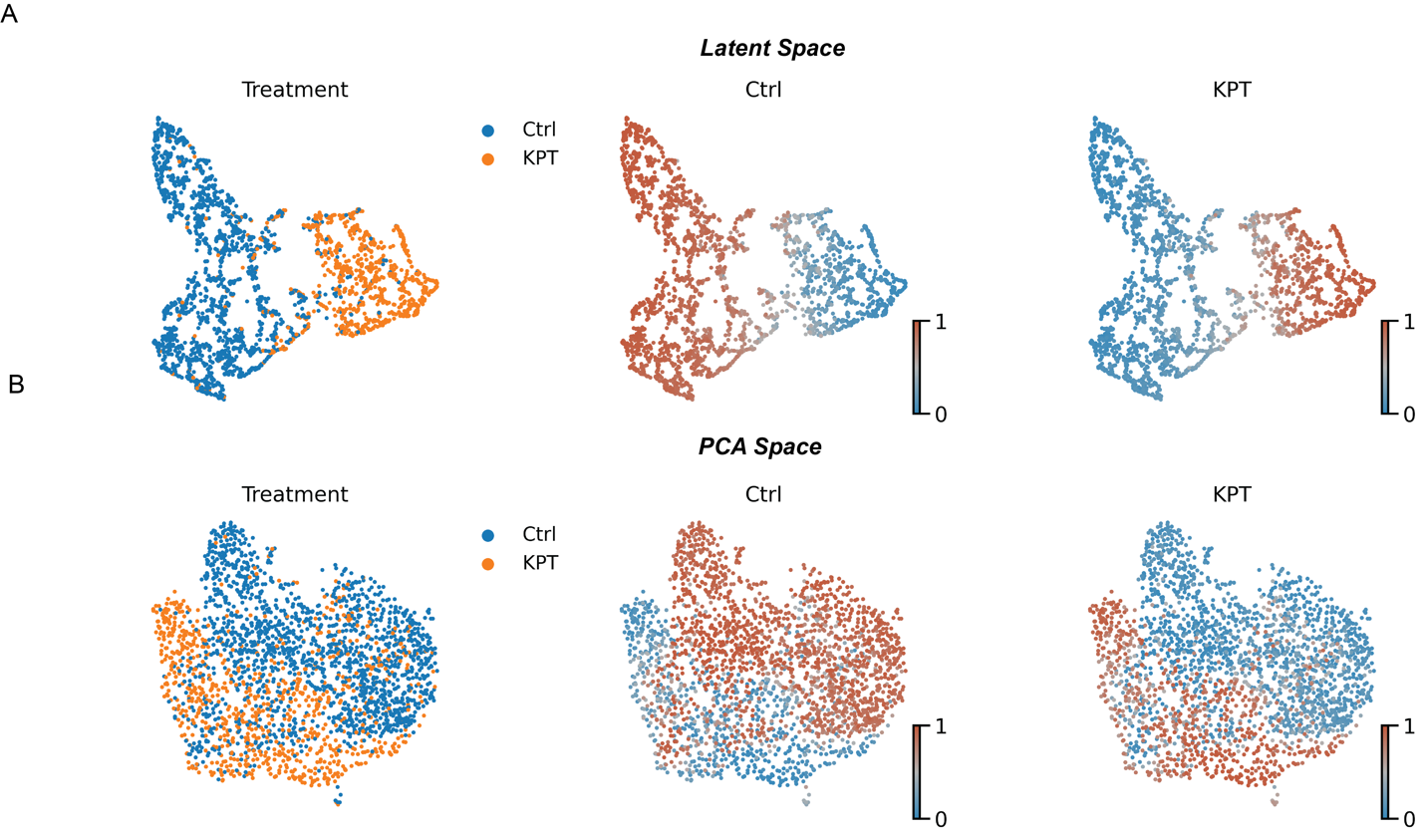


**Figure S1.** UMAP embedding of Cellograph-derived latent space trained on organoid dataset versus PCA-derived space (A) UMAP projection of the learned latent space captures visual gradients of treatment effects and separates control from KPT-treated organoids. (B) UMAP visualization on PCA coordinates yields a single globular structure with some distinction between the two treatment conditions, but does not fully capture the gradient of KPT treatment effects inferred by Cellograph.


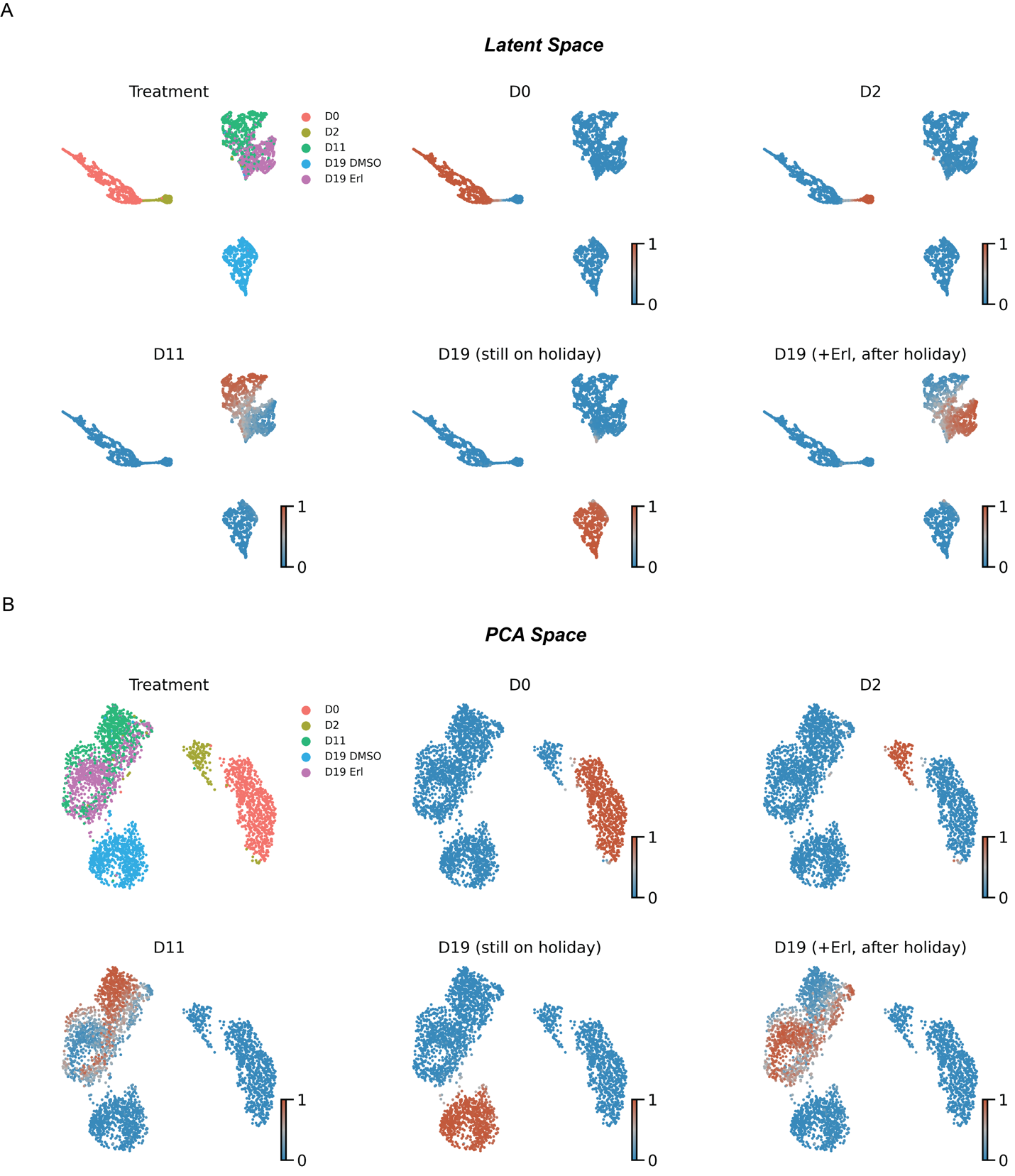


**Figure S2.** UMAP embedding of Cellograph-derived latent space trained on drug holiday dataset versus PCA-derived space (A) UMAP projection of the learned latent space captures visual gradients of treatment effects and separates the different treatment regimes compared to (B) the UMAP projection of the PCA space, which does not fully capture the gradient of the effects of Erlotinib in its axes.


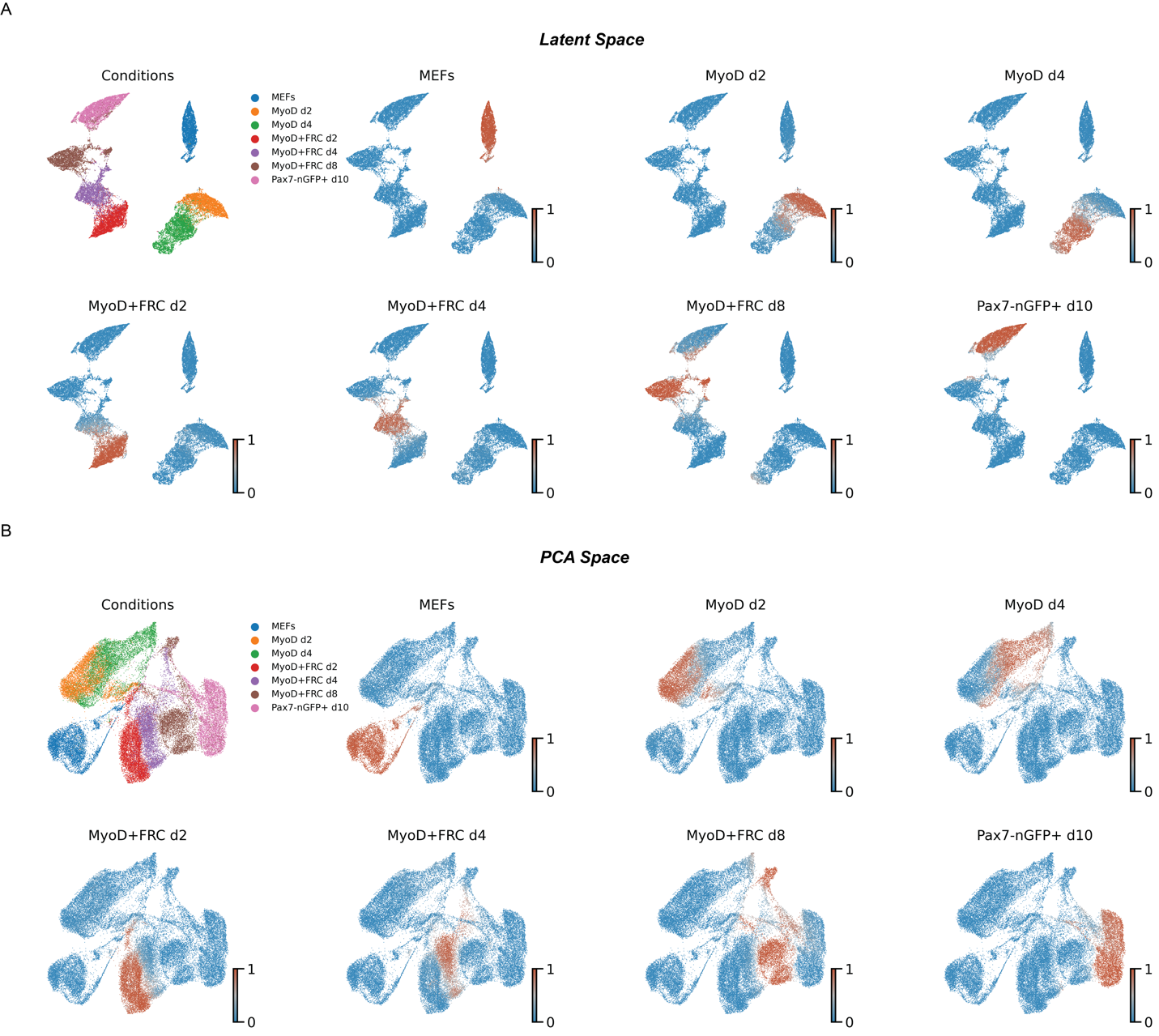


**Figure S3.** UMAP embedding of Cellograph-derived latent space trained on the myogenesis dataset versus embedding of PCA-derived space (A) UMAP projection of the learned latent space preserves the inferred visual gradients of treatment effects and separates the different differentiation stages and treatment modalities compared to (B) the UMAP coordinates computed on the PCA space, where these dynamic treatment effects are not fully captured in the visualization.
